# Is "High" Game Poisonous?

**Published:** 1889-07-13

**Authors:** 


### Within the Wards.

### IS "HIGH" GAME POISONOUS?

This question is of much wider significance than it at first appears. The workman, who never tastes game, may think he can pass it by with serene indifference as having no concern for him. But if "game" be too costly for his larder, it is not unlikely that "cold beefsteak pie" is sometimes seen there; and it is quite possible that cold beefsteak pie may now and then be as poisonous as "high" game. Doctors are terrible creatures for "investigations" and "scientific enquiries," and all that kind of thing; and occasionally it has happened that their alarming discoveries have been nothing but mares' nests. That, however, is not always the case. On the contrary, a large proportion of the "discoveries" made by medical men in recent times have taken permanent rank as verified knowledge. The poisonous character of high game will probably prove to be one of these. When meat is "tainted" there may always be found in it putrefactive microbes. These microbes, as is well known, may be killed and so rendered harmless by a sufficiently high temperature; that is to say, by a temperature which is high enough to thoroughly cook the meat. But if that be so, how can the meat still retain its poisonous properties? That is a question which has puzzled a good many thinking persons, and even doctors themselves. Dr. Lander Brunton supplies an answer which is at least plausible. He says, in effect, that the microbe may not be the only cause of putrefaction, or even the direct cause of putrefaction at all. What if the microbe secretes a poisonous substance ten times more energetic in action than itself, and what if this substance be of such a nature that heat does not destroy its deadly activity? "Meat," says Dr. Brunton, "which has become tainted by the presence of putrefactive microbes may possibly be cooked sufficiently to destroy the microbes themselves, whilst the ferments they (the microbes) have formed continue to decompose the meat and give rise to poisonous substances. We can thus see how a cold beefsteak pie, or other cold meat, may become poisonous and produce serious symptoms, although the same food may have been eaten with impunity immediately after being cooked, for during the process of slowly cooling poisons may have been formed in the meat, although there may have been none in it immediately after it had been removed from the oven, and any microbes present were likely to have been killed by the cooking. The frequency with which meat very slightly tainted must be eaten in summer, and the common rule of not eating game at all until it is somewhat "high," makes one rather wonder why poisoning by ptomaines (as the poisons produced by bacterial action are called) formed in such meat and game does not occur more frequently, although I believe that it occurs in a slight degree more frequently than people are generally willing to allow." The words "in a slight degree," really express what very often happens. There is little doubt that the simultaneous attacks of diarrhœa which seize upon several persons after partaking say of cold beefsteak pie, are to be accounted for in some such way as Dr. Brunton supposes. If it be argued that in some instances one or more individuals escape whilst others suffer from eating the very same meat or pie, the answer is, that all the portions of the pie have not reached exactly the same stage of decomposition, and that some portions may, therefore, be comparatively harmless whilst others are as decidedly poisonous. The moral of the story is, as might have been expected, that "high" game and tainted meat are undoubtedly poisonous, and that such meat should not as a rule be eaten, especially by delicate persons and children. In the summer season meat should never be kept longer than a few days; and pies should only be made of perfectly fresh steak.

